# Milk yield and composition from ewes fed diets containing narasin and their lambs’ performance

**DOI:** 10.1093/tas/txaa030

**Published:** 2020-03-12

**Authors:** Lairana A Sardinha, Rodrigo S Marques, Alexandre A Miszura, José Paulo R Barroso, Gabriela B Oliveira, André S Martins, Arnaldo C Limede, Marcos Vinicius C Ferraz Jr, Evandro M Ferreira, Alexandre V Pires, Maurice L Eastridge, Daniel M Polizel

**Affiliations:** 1 Department of Nutrition and Animal Production, FMVZ, University of São Paulo, Pirassununga, São Paulo, Brazil; 2 Elanco Animal Health, São Paulo, São Paulo, Brazil; 3 Federal University of Amazonas, Parintins, Amazonas, Brazil; 4 Department of Animal Science, “Luiz de Queiroz” College of Agriculture, University of São Paulo, Piracicaba, São Paulo, Brazil; 5 Department of Animal Science, The Ohio State University, Columbus, OH

**Keywords:** additives, feed efficiency, glucose, milk production, sheep

## Abstract

The changes promoted by feed additives in ruminal fermentation, especially increasing the availability of propionate, can improve the energy balance of an animal, which is of great importance in the lactation period. This trial aimed to evaluate the inclusion of narasin in the diet of lactating ewes on milk yield, composition, dry matter intake (DMI), and plasma metabolites of the ewes and growth rate of lambs. Thirty-two lactating ewes (59.0 ± 2.42 kg) were assigned to a randomized complete block design. The experimental diets contained 500 g/kg of dry matter (DM) of coast cross (*Cynodon dactylon* (L.) Pers) hay and 500 g/kg DM of concentrate, and the treatments were: N0—no narasin inclusion; N13—inclusion of 13 mg of narasin/kg DM. Once a week, from week 2 to 10 of lactation, ewes were separated from their lambs, injected with oxytocin, and milked mechanically to empty the udder. After 3 h, the milk production was recorded, using the same procedure, and sampled to evaluate the composition. The blood samples were taken weekly, 4 h after feeding. The average daily gain (ADG) and starter DMI of the lambs were evaluated weekly from week 2 to 12 of age. The inclusion of narasin did not affect (*P* = 0.93) DMI of ewes; however, it increased milk production (*P* < 0.01) and feed efficiency (*P* = 0.02; FE). Ewes fed N13 had a greater milk fat (*P* < 0.01), protein (*P* < 0.01), lactose (*P* = 0.04), and total solids production (*P* < 0.01). Narasin inclusion in ewe’s diet increased plasma glucose concentration (*P* = 0.05) at weeks 8, 9 and 10; however, there was no effect on plasma urea concentration (*P* = 0.96). The lambs of N0 ewes had a greater starter DMI (*P* < 0.01) at weeks 7, 8, 9, and 10; however, the ADG and body weight at weaning and after weaning were similar between treatments (*P* > 0.05). The results showed that the inclusion of 13 mg of narasin/kg DM improved the milk production and FE of the ewes without altering the composition of the milk. The lower initial consumption of concentrate by N13 lambs before weaning was caused by the higher production of milk. The results obtained in the present study demonstrate the possible productive gain with the inclusion of narasin in diets for lactating ewes.

## INTRODUCTION

The survival and development of lambs are directly linked to the production of milk since this is the first food ingested by the newborn. It is common to use feed management strategies to improve milk composition and production in an effort to optimize lamb productivity and carcass quality. To that end, the feeding with additives is of interest for increasing milk production of ewes.

The changes promoted by ionophores in ruminal fermentation, further increasing the availability of propionate, result in improvements in energy balance because the increase of the molar concentration of propionate increases hepatic gluconeogenesis, contributing to the increase of milk production (Oba and Allen, 2003). Monensin and lasalocid are the most studied ionophores, and their use in dairy cows increased energy status during the transition and early lactation periods (McGuffey et al., 2001).

Narasin is an ionophore produced by *Streptomyces aureofaciens* bacteria, with a molecular formula of C_43_H_72_O_11_ and is soluble in alcohol, acetone, chloroform, and ethyl acetate (Berg and Hamill, 1978). The ability of narasin to carry ions through cell membranes made it possible for tests of population control of bacteria. In an in vitro study, Nagaraja et al. (1987) observed that narasin increased the molar concentration of propionate with lower doses in relation to monensin and lasalocid and was more effective in the decrease in the production of lactic acid than the other additives evaluated. Narasin inclusion in diets for feedlot lambs increased the average daily gain (ADG), feed efficiency (FE), and final body weight (BW; Polizel et al., 2016). However, the effects of narasin on milk production, milk composition, and plasma parameters in lactating ewes have not been described.

In this context, we hypothesized that the inclusion of narasin in the diet of ewes postpartum will increase milk production and lamb performance without changing milk composition and dry matter intake (DMI). Hence, the objective of the present study was to evaluate the inclusion of narasin in diets for lactating ewes on DMI, milk production and composition, plasma metabolites of the ewes, and growth of suckling lambs.

## MATERIALS AND METHODS

This study was carried out at sheep facilities of the Department of Animal Science, “Luiz de Queiroz” College of Agriculture, University of São Paulo, Piracicaba, São Paulo, Brazil. All procedures using animals followed the guidelines recommended by the Animal Care and Use Committee of the University of São Paulo (protocol number 5020291118).

### Animals and Experimental Design

Thirty-two Dorper × Santa Inês ewes with initial BW of 59.0 ± 2.42 kg were housed indoor and individually allotted with their lambs in pens (1.5 × 3.5 m) with a concrete floor, feed bunk, mineral box, waterer, and a creep feeding system (0.80 × 1.0 m). Eighteen ewes had single births (9 per treatment) and 14 had twin births (7 per treatment), with 18 females (9 per treatment) and 28 male lambs (14 per treatment). At lambing, all ewes were dewormed with moxidectin 1% (Cydectin, Fort Dodge Saúde Animal Ltda, Campinas, São Paulo, Brazil) according to label directions.

After 7 ± 1.2 d in milk, ewes were divided into a randomized complete block design, with two diets and 16 blocks (*n* = 32). Within blocks (*n* = 10), ewes were randomly assigned to the treatments. The blocks were defined according to date of lambing, type of birth, sex of the offspring, and initial BW of the ewes. Four blocks included ewes nursing single female lambs; five blocks included ewes nursing single male lambs; and seven nursing twins (four male–male, two female–female, and 1 male–female). The experimental period was from 2 to 10 wk postpartum for ewes, and their lambs were evaluated until 12 wk of age.

### Food Management, Collection of Samples, and Methodologies

After forming the blocks, ewes were fed the control diets containing 500 g/kg of dry matter (DM) of coast cross (*Cynodon dactylon* (L.) Pers) hay, and 500 g/kg DM of concentrate, to adapt them to the experimental facilities and feeding management. The treatments were: N0—control diet with no fed additives; and N13—inclusion of 13 mg of narasin/kg DM (ZIMPROVA, Elanco Animal Health, Indianapolis, IN). The dose used was determined according to previous studies (Assis et al. 2019). The diets (Table 1) were isonitrogenous and balanced according to the recommendations of the National Research Council (NRC 2007).

**Table 1.** Proportions of the ingredients and chemical composition of the experimental diets

	Diets^*a*^	
Item	N0	N13
Ingredients, %		
Hay “coast cross”	50.0	50.0
Soybean meal	8.6	8.6
Citrus pulp	18.2	18.2
Ground corn	18.2	18.2
Mineral mix^*b*^	1.5	1.5
Soybean oil	3.0	3.0
Urea	0.5	0.5
Narasin, mg/kg of DM	0	13.0
Chemical composition, %		
DM, as-fed basis	88.5	88.5
Organic matter	92.0	92.0
CP	13.9	14.1
NDF	46.0	46.1
ADF	20.4	20.5
EE	4.5	4.6
Ash	8.0	8.0
NFC	28.5	28.1

^*a*^N0 = diet without feed additive; N13 = diet containing 13 mg/kg of narasin of DM.

^*b*^Ca: 22%, P: 5.5%, Mg: 3.5%, S: 2.2%, Cl: 10.55%, Na: 7.0%, Mn: 1500 mg/kg, Fe: 500 mg/kg, Zn: 1550 mg/kg, Cu: 440 mg/kg, Co: 50 mg/kg, I: 40 mg/kg, and Se: 20 mg/kg.

The concentrate ingredients were weighed using an electronic scale with a 10-g accuracy (Marte, LC 100 São Paulo, Brazil) and mixed using a horizontal mixer with a capacity of 500 kg (Lucato, Limeira, Brazil). The hay was ground using a shredder (Nogueira DPM—4, Itapira, São Paulo, Brazil) with a 10-mm screen. Narasin was previously mixed with the concentrate (Lucato, Limeira, Brazil) and supplied to the sheep as a total mixed ration. The animals had ad libitum access to the feed and freshwater. The diet was offered daily and orts were collected and weighed weekly in order to calculate DMI. Amounts of feed offered to ewes were calculated according to previous DMI, and adjustments were done when needed so that refused feed did not exceed 0.1 kg of daily intake. Feeds and orts were sampled weekly and frozen at −20 °C for later analysis.

Ewes were weighed without fasting for three consecutive days at the beginning and at the end of the experimental period. The initial BW and final BW were determined as the average of the three-weight data. On the days of weighing, the body condition score (BCS) was also assessed by classifying the ewes with grades from 1 (thin) to 5 (fat), with an increment of 0.25 (Russel et al., 1969).

To measure the milk production, once a week (weeks 2–10), the ewes were separated from their lambs and mechanically milked (Camp Agri, model GL300, São Paulo, Brazil) twice a day after an intravenous injection of 10 IU of synthetic oxytocin (Univet, São Paulo, Brazil) at 1000 and 1300 h (Susin et al., 1995). The first milking was performed to empty the mammary gland and the milk was discarded. The second one was used to measure milk yield in 3 h. The total milk produced per ewe in this interval was weighed on an electronic scale accurate to 0.1 g (Marte AC-10K). After weighing, milk was homogenized and 20 mL was collected and preserved in bromopol Broad Spectrum Microtabs II (2-bromo-2-nitropropane-1.3-diol, D & F Control Systems, Inc., Dublin, CA).

From 2 to 10 wk of lactation, blood samples were collected weekly at 4 h after offering the diet from the ewe’s jugular vein into Vacutainer tubes containing sodium fluoride as a glycolytic inhibitor and the anticoagulant Ethylenediaminetetraacetic acid (Greinr Bio-One Brazil, Americana, São Paulo, Brazil). Immediately after drawing, blood samples were centrifuged at 3,000 × *g* at 4 °C for 15 min. After centrifugation, two aliquots were obtained from the plasma and were stored separately at −18 °C, which were then used to determine the concentration of glucose and urea. The determinations of plasma glucose and urea were performed using specific commercial enzymatic kits from LABTEST diagnostic S.A. (Lagoa Santa, Minas Gerais, Brazil; Ref.: 85) in an automatic biochemistry system (SBA—200, CELM, Barueri, São Paulo, Brazil).

### Lamb Performance

Lambs had ad libitum access to feeding starter from week 2 to 12 of age. The starter was composed by 645 g/kg DM ground corn, 190 g/kg DM soybean meal, 5 g/kg DM ammonia chloride, 15 g/kg DM mineral mix, 5 g/kg DM limestone, 40 g/kg DM molasses, and 100 g/kg DM milk replacer. The starter was available in a creep feeding system (0.80 × 1.0 m). To avoid access to the ewes’ feed bunk, lambs were kept in a leash system, allowing them to nurse and to reach the creep feeder and water (Polizel et al., 2017; Parente et al., 2018). The orts of creep feeding were weighted weekly on an electronic scale with a 1-g accuracy (Marte, LC 100, São Paulo, Brazil) for DMI calculations. Lamb BW was measured weekly after a 3-h fast to calculate the ADG. Lamb DMI was obtained during the preweaning (14–70 d of age) and postweaning (70–84 d of age) periods.

### Chemical Analysis

The samples of feed and orts were dried (MA035—Marconi, Piracicaba, São Paulo, Brazil) at 55° for 72 h and ground through a 1-mm Wiley mill (Marconi, Piracicaba, Brazil). The DM was determined by drying the samples in an oven at 105 °C for 24 h (Association of Official Agricultural Chemists; AOAC, 1990; #934.01), and ash was determined by incinerating the samples in muffle at 550 °C for 4 h (AOAC, 1990; #942.05). Sequential detergent fiber analyses were used to determine neutral detergent fiber (NDF; Van Soest et al., 1991) and acid detergent fiber (ADF; Goering and Van Soest, 1970) concentrations in an Ankom 2000 fiber analyzer (Ankom Tech. Corp., Fairport, NY). Heat-stable α-amylase and sodium sulfite were included in the NDF analysis. Total nitrogen concentration was determined using the Leco TruMac N apparatus (Leco Corporation, St. Joseph, MI; AOAC, 1990; #968.06). The crude protein (CP) was calculated by multiplying the total nitrogen by 6.25. The ether extract (EE) concentration was determined according to AOAC (1990; #920.29). Nonfiber carbohydrates (NFC) of the diets were estimated according to the following equation: NFC = 100 − [(%Total CP − %CP urea + % urea) + %neutral detergent fiber + %EE + %ash] (Hall, 2000).

Milk samples were analyzed for protein, fat, lactose, and total solids. Fat, protein, and lactose concentrations were determined by infrared spectrometry (Bently 2000; Bently Instruments Inc., Chasca, MN; AOAC, 1990). Milk correction calculations for fat (6.5%; fat-corrected milk, FCM) and protein (5.8%; fat- and protein-corrected milk, FPCM) were performed as described by Pulina and Nudda (2002). The FE was calculated for milk production, FCM and FPCM considering the DMI each week.

### Statistical Analysis

Pen was the experimental unit for all statistical analyses. Statistical analyses were performed using the MIXED procedure of SAS (SAS version 9.0; SAS Inst. Inc., Cary, NC). All data were submitted to the Shapiro–Wilk test to verify the normality of the residuals, the removal of “outliers” using the studentized residuals, and homogeneity of variances using the Levene test.

The data for intake, milk production, milk composition, performance of the lambs, and plasma metabolites were analyzed as repeated measures over time. The statistical model used was: *y*_*ijk*_*= μ + D*_*i*_*+ b*_*j*_*+ e*_*ij*_*+ T*_*k*_*+ D*_*i*_*T*_*k*_*+ b*_*j*_*T*_*k*_*+ e*_*ijk*_, in which *μ* = overall mean, *D*_*i*_ = fixed effect of diet, *b*_*j*_ = random block effect, *e*_*ij*_ = random error A, *T*_*k*_ = fixed effect of time, *D*_*i*_*T*_*k*_ = fixed effect of diet × time interaction, *b*_*j*_*T*_*k*_ = random effect of block × time interaction, and *e*_*ijk*_ = random error B. All analyzed data as repeated measures were put on covariance matrices and tested for “compound symmetry, heterogeneous compound symmetry, autoregressive, autoregressive heterogeneous, unstructured, banded, variance components, toeplitz, and heterogeneous toeplitz” and defined according to the lowest value obtained for Akaike’s information criterion. The treatment means were obtained by the LSMEANS command. The effect of diet, time, and interaction of diet × time were defined by the *F* test.

Ewe BW, BCS, and BW of lambs were analyzed using the model *y*_*ij*_*= μ + D*_*i*_*+ b*_*j*_*+ e*_*ij*_, in which *μ* = overall mean, *D*_*i*_ = fixed effect of diet, *b*_*j*_ = random effect of block, and *e*_*ij*_ = random error. The treatment means were obtained by the LSMEANS command. The treatment effect was defined by the *F* test. All analyzed variables were considered significant when *P* < 0.05.

## RESULTS

The experimental diets did not affect the BW (*P* = 0.52) and BCS (*P* = 0.81) of ewes at week 10 of lactation (Table 2). There was no interaction between diets and week on DMI (*P* = 0.13), nutrient intake, milk yield (*P* = 0.41), milk composition, and FE of ewes (Table 2). The inclusion of narasin increased milk production (*P* < 0.01). Moreover, narasin did not affect the milk composition and, consequently, increased FCM (*P* = 0.01), FPCM (*P* = 0.01), fat (*P* < 0.01), protein (*P* < 0.01), lactose (*P* = 0.04), total solids (*P* < 0.01), and solids nonfat production (*P* < 0.01). The narasin inclusion increased the FE for milk (*P* = 0.02), FCM (*P* = 0.02), and FPCM (*P* = 0.02) compared with the control diet.

**Table 2.** BW, BCS, milk production, and composition of the ewes fed experimental diets

	Diets^*a*^			*P*-value		
Item	N0	N13	SEM	Diet (D)	Time (T)	D × T
BW, kg						
Week 2	58.4	59.7	2.42	0.58	—	—
Week 10	57.8	59.4	2.11	0.52	—	—
BCS						
Week 2	2.83	2.88	0.07	0.62	—	—
Week 10	3.10	3.13	0.09	0.81	—	—
Intake, kg/d						
DM	2.27	2.26	0.10	0.93	<0.01	0.13
CP	0.30	0.32	0.02	0.23	<0.01	0.14
NDF	1.04	1.03	0.05	0.99	<0.01	0.18
ADF	0.46	0.46	0.02	0.80	<0.01	0.24
Ash	0.18	0.18	0.01	0.98	<0.01	0.29
Production, g/3 h						
Milk	194	241	15.5	<0.01	<0.01	0.41
FCM	235	314	24.4	0.01	<0.01	0.48
FPCM	219	292	22.3	0.01	<0.01	0.49
Fat	16.5	22.4	1.83	<0.01	0.04	0.23
Protein	7.95	10.03	0.64	<0.01	<0.01	0.72
Lactose	9.33	11.14	0.97	0.04	<0.01	0.43
Total solids	35.2	45.9	3.38	<0.01	0.02	0.16
Solids nonfat	19.1	23.5	1.57	<0.01	<0.01	0.38
Composition						
Fat, %	8.65	9.23	0.33	0.21	<0.01	0.98
Protein, %	4.13	4.16	0.10	0.79	<0.01	0.06
Lactose, %	4.78	4.66	0.06	0.19	<0.01	0.70
Total solids, %	18.5	19.0	0.23	0.25	<0.01	0.94
Solids nonfat, %	9.88	9.73	0.09	0.17	<0.01	0.26
Urea, mg/dL	16.0	14.9	0.87	0.40	<0.01	0.91
Somatic cell count	1434	1668	334	0.28	0.41	0.54
FE						
Milk/DMI	0.69	0.94	0.08	0.02	<0.01	0.28
FCM/DMI	0.85	1.20	0.10	0.02	0.01	0.57
FPCM/DMI	0.78	1.11	0.09	0.02	0.02	0.43

^*a*^N0 = diet without feed additive; N13 = diets containing 13 mg/kg of narasin of DM.

There was a time effect (*P* < 0.01) on intakes of DM, CP, NDF, ADF, and ash, with a progressive increase and the highest values found at week 7 of lactation, followed by a decrease. There was a time effect (*P* < 0.01) on milk production and composition. The milk, FCM, FPCM, protein, lactose, total solids, and solids nonfat production was greater at week 4, followed by a progressive decrease until week 10 of lactation (*P* < 0.01). The percentage of fat, protein, total solids, and solids nonfat was greater at week 10; however, the lactose percentage was greatest at weeks 4 and 5. The FE for milk production, FCM, and FPCM decreased during the lactation, with the greatest values observed at week 3.

**Figure 1.**
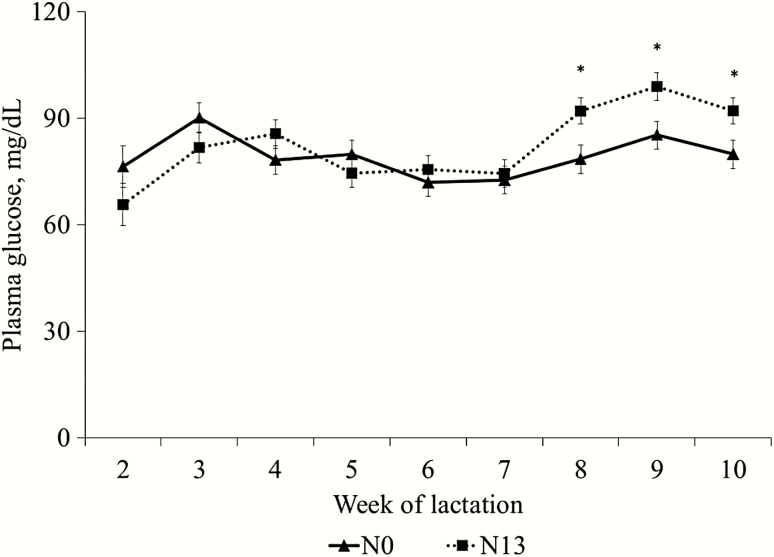
Plasma glucose concentration in ewes fed the control diet (no additive; N0) or a diet containing 13 mg/kg of narasin of DM (N13) from samples collected from week 2 to 10 related to lambing. There was a diet × time effect (*P* = 0.01) for plasma glucose concentration. Diet containing narasin increased (*P* < 0.01) glucose concentration in ewes at weeks 8, 9, and 10.

**Figure 2.**
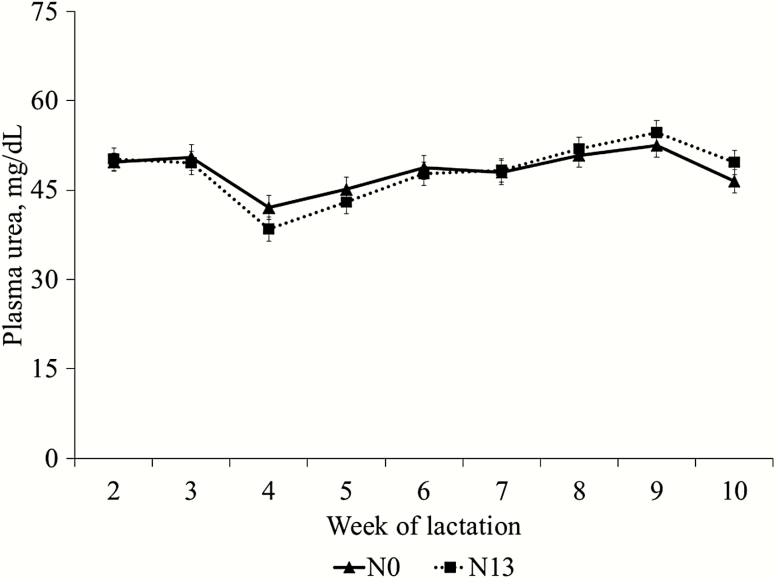
Plasma urea concentration in ewes fed the control diet (no additive; N0) or a diet containing 13 mg/kg of narasin of DM (N13) from samples collected from week 2 to 10 related to lambing. There was no diet and week interaction (*P* = 0.60) and diet effect (*P* = 0.96) for plasma urea. There was a time effect (*P* < 0.01) with the greater values observed on week 9 of lactation.

There was an interaction (*P* < 0.01) between diets and week for concentration of glucose (Fig. 1). The narasin inclusion did not affect the plasma glucose concentration at the weeks 3, 4, 5, 6, and 7; however, the ewes fed the N13 diet had a greater plasma glucose concentration (*P* = 0.05) than N0 at weeks 8, 9, and 10. There was no interaction (*P* = 0.60) between diets and week and diet effect (*P* = 0.96) for plasma urea; however, there was a time effect (*P* < 0.01) with the greater values observed at week 9 of lactation (Fig. 2).

**Figure 3.**
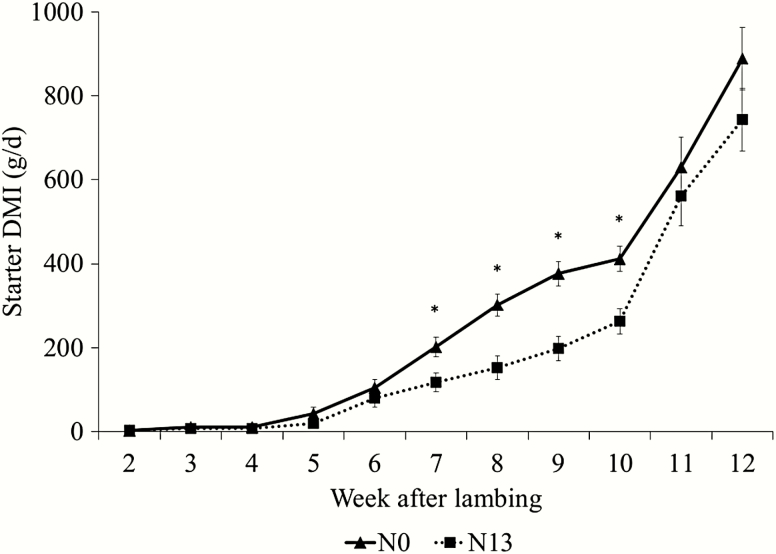
Starter DMI (g/d) of lambs from ewes fed the control diet (no additive; N0) or ewes fed diet containing 13 mg/kg of narasin of DM. There was a diet × time interaction (*P* = 0.03) for starter DMI before weaning. Lambs from ewes fed N13 had a lower starter DMI (*P* < 0.02) at weeks 7, 8, 9, and 10 related to lambing.

The experimental diets did not affect the lamb ADG (*P* > 0.05); consequently, BW at weaning and after weaning was similar among treatments (*P* > 0.05; Table 3). However, there was an interaction (*P* = 0.03) between diets and week for initial concentrate DMI by the lambs before the weaning. The lambs had the same initial concentrate DMI at weeks 3, 4, 5, and 6; however, lambs from N0 ewes had a greater (*P* < 0.05) initial concentrate DMI compared with N13 at weeks 7, 8, 9, and 10 (Fig. 3). There was a time effect *(P* < 0.01) for ADG before and after weaning, with the greater values observed at weeks 8 and 11, respectively. The initial concentrate DMI increased during the lactation of the ewes and after weaning.

**Table 3.** BW, ADG, and DMI of lambs from ewes fed experimental diets

	Diets^*a*^			*P*-value		
Item	N0	N13	SEM	Diet (D)	Time (T)	D × T
BW, kg						
Week 2	6.86	6.86	0.35	0.98	—	—
Weaning	22.1	21.2	1.03	0.35	—	—
After weaning^*b*^	26.3	25.7	1.11	0.60	—	—
ADG, g						
Before weaning	263	266	13.8	0.77	<0.01	0.23
After weaning^*b*^	296	294	24.1	0.95	<0.01	0.28
Starter DMI, g/d						
Before weaning	186	106	20.21	<0.01	<0.01	0.03
After weaning^*b*^	759	652	67.7	0.19	<0.01	0.30

^*a*^N0 = diet without feed additive; N13 = diets containing 13 mg/kg of narasin of DM.

^*b*^After weaning represents the average of 14 d after weaning.

## DISCUSSION

### Ewes DMI, BW, milk yield, and composition

The DMI was 2.27 and 2.26 kg/d for ewes fed N0 and N13 diets, respectively. These are above the values recommended by the NRC (2007) for 60 kg ewes rearing single or twin lambs during the first 6–8 wk of lactation. Differently than monensin (Duffield et al., 2012), some research shows that the narasin inclusion on ruminant diets did not affect DMI in high forage diets (Silva et al., 2015; Polizel et al., 2019) and in high concentrate diets (Gobato et al., 2017; Polizel et al., 2016) as in the present study. The literature has demonstrated that the inclusion of narasin in the diet has increased the FE of the animals with increase in performance, without reducing the DMI.

As there was no effect on DMI of the ewes during the experiment period, the consumption of NDF, ADF, and ash was equal for the animals of both experimental diets. In this experiment, the BW and BCS were similar during the experimental period, showing that the animals did not need to perform energy mobilization to maintain milk production.

Narasin improves milk production when compared with control diets. The apparent benefits of ionophore feeding in the lactation period are linked to a better glucose state, caused by increased production of propionate and better retention of nitrogen (McGuffey et al., 2001). The effect on FE for milk, FCM, and FPCM of the N13 diet is due to the improved milk production of these animals and to the similar dietary intake of the N0 diet. Narasin is capable of changing rumen fermentation, especially to increase the molar proportion of propionate and decrease the acetate:propionate ratio (Polizel et al., 2019). The increase in propionic acid concentrations increases gluconeogenesis in hepatic tissue and improvement in energy metabolism (Baird et al., 1980). It generates an improvement in the efficiency of retention of crude energy by the animal since propionate is the main precursor of glucose for ruminants.

With the advancement of lactation, the epithelial cells of the mammary gland pass from the state of active secretion to the nonsecretory state by the involution process, characterizing the decline of production at the end of lactation (Pulina and Nudda, 2002). There was an effect (Table 2) on the production of fat, protein, lactose, total solids, and solids nonfat since the production of these milk compounds accompanies milk production. There was no effect of dilution of the milk compounds when there was an increase in milk production of the animals during the experimental period. As described by Resende et al. (2008), the period of lactation, from a nutritional point of view, is one of the moments that should be more focused on females since they are faced with three distinct phases. In the first, shortly after the birth of the lamb, the ewe goes through a negative energy balance since the milk production of this animal is increasing and its consumption has not yet reached the maximum potential; thus, the mobilization of body reserves takes place. In the second phase, the energy balance is equal to zero since milk production is already decreasing and the female has already reached the peak of DM consumption. In the last phase, the energy balance is positive, with the replacement of the body reserves. Therefore, the present study demonstrated that narasin provided a greater energy supply to the animals, with this energetic gain destined to the production of milk and also of the milk compounds without alterations in milk composition.

### Plasma Metabolites

The increase in plasma glucose from week 7 to 10 of lactation in ewes fed diets containing narasin can be explained by the energy balance of the ewes at the end of lactation, resulting in a decrease in milk production. The inclusion of narasin in ruminant diets increases molar proportion of propionate (Polizel et al., 2019), and propionate is the main glucose precursor in ruminants (Ellis et al., 2012), which at the end of lactation was shown at higher levels due to the lower glucose requirement of these animals.

There was no effect on plasma concentrations of urea during the experiment. This is due to the fact that there is a relation between plasma urea concentrations and the amount of energy consumed (Cardoso et al., 2010) since urea levels may be associated with protein levels in the diet and also reflect the energy and protein ratio of the diet. In the present study, the treatments did not affect the consumption of DMI and protein, resulting in the same plasma urea concentration.

The main controlling factor of plasma urea concentrations is the formation of ammonia in the rumen, and the concentration of plasma urea appears to reflect changes in rumen ammonia production. Thus, the concentration of urea in the plasma is influenced by the extent to which the absorbed amino acids are oxidized and by the absorption of ammonia from the rumen, substantially reflecting the extent of nitrogen balance in the animal, considering both the requirements of ruminal microorganisms and of the host animal (Lima, 2013). Ionophores, such as monensin and narasin, are potential feed additives to decrease rumen ammonia, especially to inhibit deamination (Bergen et al., 1984), which could decrease plasma urea concentration; however, this was not observed in the present study.

### Lamb Performance

Even with the higher N13 milk production in relation to N0, there was no effect on ADG and BW of the lambs of both treatments. The ADG of lambs during lactation was higher than in one of our previous studies (Ferreira et al., 2018) using the same methodology but presented values ​​close to those reported by Susin et al. (1995). The lack of effect on ADG and BW of lambs is likely explained by the greater DMI of lambs born to ewes assigned to N0 treatment. The consumption of food in the trough is inversely proportional to the amount of milk ingested (Foot, 1972; NRC, 2007) or it also results in the suppression of constant suckling (Folman et al., 1966). There is a correlation between offspring milk consumption and growth rate of these lambs (Roda et al., 1984). This correlation is high during the first 6 wk of lactation, and then declines and may become negative at the later stage of lactation (Peart, 1982).

The decline in the lactation curve is impacted by the decrease in milk demand by lambs due to increased solid food intake. Barnicoat et al. (1949) found that milk loses importance from week 8 of age of lambs, as they increase grain and forage consumption. In the present study, the young lambs started to really increase the consumption of solid foods at around day 21 of age.

Suckling lambs that receive the least amount of milk increase the intake of solid foods more quickly in early life than those lambs that receive the most amount of milk at that stage. A lamb meets its energy through increased food intake, and weight gain, in this case, is influenced by the consumption of solid foods during the suckling of these animals (Hatfield et al., 1995).

Therefore, lambs that had greater access to higher amounts of milk, as in the N13 treatment, did not consume a large amount of concentrate in creep feeding when compared with lambs with lower milk yield. The higher milk content available for lambs of narasin tended to supply the energy needs, reducing the need for diets with solids consumption as a function of the satiety generated by milk, reducing the need for concentrate consumption. After weaning, the consumption of starter grain of these animals was similar due to the absence of milk.

In summary, milk production is known as one of the limiting factors for lamb growth. The narasin inclusion (13 mg/kg of DM) in the diet of lactating ewe’s increased FE and milk production without altering its composition, making the lamb less dependent on the initial concentrate since its requirements for gain are mainly attended by the ewes’ milk yield. In addition, the inclusion of narasin increased plasma glucose concentration after the peak of lactation, providing more energy to the ewes. The results obtained in the present study demonstrate the possible productive gain with the inclusion of narasin.
